# Cutaneous Manifestations in Autoimmune Polyendocrinopathy-Candidiasis-Ectodermal Dystrophy (APECED): A Comprehensive Review

**DOI:** 10.3390/biomedicines12010132

**Published:** 2024-01-09

**Authors:** Florica Sandru, Razvan-Cosmin Petca, Mihai Cristian Dumitrascu, Aida Petca, Andreea-Iuliana Ionescu (Miron), Livia-Cristiana Baicoianu-Nitescu

**Affiliations:** 1 Department of Dermatovenerology, “Carol Davila” University of Medicine and Pharmacy, 020021 Bucharest, Romania; florica.sandru@umfcd.ro (F.S.); livia.nitescu@rez.umfcd.ro (L.-C.B.-N.); 2 Dermatology Department, “Elias” University Emergency Hospital, 011461 Bucharest, Romania; 3 Department of Urology, “Carol Davila” University of Medicine and Pharmacy, 050474 Bucharest, Romania; 4 Department of Urology, ‘Prof. Dr. Th. Burghele’ Clinical Hospital, 050659 Bucharest, Romania; 5 Department of Obstetrics and Gynecology, “Carol Davila” University of Medicine and Pharmacy, 050474 Bucharest, Romania; 6 Department of Obstetrics and Gynecology, University Emergency Hospital of Bucharest, 050098 Bucharest, Romania; 7 Department of Obstetrics and Gynecology, “Elias” University Emergency Hospital, 011461 Bucharest, Romania; 8 Department of Oncological Radiotherapy and Medical Imaging, “Carol Davila” University of Medicine and Pharmacy, 020021 Bucharest, Romania; 9 Department of Medical Oncology, Colțea Clinical Hospital, 030167 Bucharest, Romania

**Keywords:** APS-1, APECED, mucocutaneous candidiasis, vitiligo, alopecia areata, cutaneous manifestations, classic triad, chronic hypoparathyroidism, Addison’s disease

## Abstract

Autoimmune polyendocrinopathy-candidiasis-ectodermal dystrophy (APECED), or polyglandular autoimmune syndrome type 1 (PAS-1/APS-1), is a rare autosomal recessive disorder linked to mutations in the autoimmune regulator (AIRE) gene. This review provides a detailed analysis of cutaneous manifestations in APECED, focusing on chronic mucocutaneous candidiasis (CMC), alopecia areata (AA), and vitiligo. The classic triad of hypoparathyroidism, adrenal insufficiency, and CMC serves as a diagnostic cornerstone. However, the varied clinical spectrum of APECED, particularly its cutaneous presentations, poses a diagnostic challenge. CMC, often an early sign, varies in prevalence across populations, including Finnish (100%), Irish (100%), Saudi Arabian (80%), Italian (60–74.7%), North American (51–86%), and Croatian (57.1%) populations. Similarly, AA prevalence varies in different populations. Vitiligo also exhibits variable prevalence across regions. The review synthesizes the current knowledge arising from a narrative analysis of 14 significant human studies published in English up to October 2023. Moreover, this paper underscores the importance of early detection and monitoring, emphasizing cutaneous manifestations as key diagnostic indicators. Ongoing research and clinical vigilance are crucial for unraveling the complexities of this rare autoimmune syndrome and enhancing patient care.

## 1. Introduction

Polyglandular autoimmune syndrome type 1 (PAS-1/APS-1), also known as autoimmune polyendocrinopathy candidiasis-ectodermal-dystrophy (APECED), is a rare autosomal recessive disorder. It has an average incidence of 10 cases per million inhabitants and it occurs due to mutations in the autoimmune regulator (AIRE) gene. Diagnosis generally relies on three main criteria: chronic mucocutaneous candidiasis (CMC), chronic hypoparathyroidism (CH), and adrenal insufficiency. Nonetheless, genetic testing is necessary for precise identification in certain instances, particularly those presenting with atypical or subtle symptoms [1,2,3].

In addition to the typical manifestations, individuals with APECED may also manifest various autoimmune conditions, including but not limited to autoimmune hepatitis, enteropathy, gastritis, pernicious anemia, gonadal failure, and diabetes. Furthermore, the syndrome is linked to ectodermal manifestations such as alopecia and vitiligo, along with inflammatory complications such as intestinal lung disease and nephritis [2,3,4,5].

The prognosis for individuals with APECED is intricate and contingent on several factors. Genetic elements, encompassing mutations in the AIRE gene and particular human leukocyte antigen (HLA) associations, coupled with hormonal imbalances and environmental factors such as infections, may significantly influence the progression of the disease [3,5]. 

Chronic mucocutaneous candidiasis (CMC) can manifest in two ways: isolated CMC or syndromic CMC, associated with other clinical manifestations, mainly infectious and autoimmune conditions [6,7]. Over the past 13 years, research has emphasized the significance of IL-17-mediated immunity in protecting against mucocutaneous candidiasis. Investigations into syndromic CMC have revealed insights, such as the role of IL-17A/F in safeguarding against *Candida* spp., in patients with specific genetic mutations such as dominant-negative signal transducer and activator of transcription 3 (STAT3), hyper-IgE syndrome, caspase recruitment domain-containing protein 9 (CARD9) deficiency, IL-12p40 deficiency, or IL-12Rβ1 deficiency. These conditions result in reduced IL-17A/F-producing T cells, suggesting the importance of IL-17A/F in mucocutaneous defense against *Candida* spp. [8,9,10]. In the case of patients with autoimmune polyendocrine syndrome type 1 (APS-1), they exhibit thymic escape of autoreactive T cells and the early development of various autoantibodies against different autoantigens, including tissue-specific antigens and specific cytokines [6,11]. Notably, the onset of APS-1 has been reported to occur within the first year of life in a significant percentage of patients (24–52%) [12,13].

Vitiligo, a common disorder of pigmentation and true depigmentation, is estimated to affect 0.5% to 2% of the global population [14]. The disappearance of vitiligo melanocytes involves various mechanisms such as necrosis, necroptosis, pyroptosis, and apoptosis. Elevated oxidative stress damages melanocytes and induces autoimmunity through the upregulation of calreticulin expression and translocation to the cell membrane. Cytokines, including IL-1, IL-6, TNF-α, and possibly IL-17, play a role in vitiligo pathogenesis [15,16]. A meta-analysis of 17 studies involving 4365 subjects, predominantly women, conducted by Farajzadeh et al., revealed varying prevalence rates for non-segmental vitiligo types, with vulgaris, focal, and acrofacial being the most common. Moreover, positive family history varied by region from 13.91% (Asia with 11 studies) to 27.01% (Europe with two studies), and phenomena such as Kobner phenomena and leukotrichia were noted in a significant percentage of patients [17].

Alopecia areata (AA) is an autoimmune condition characterized by T-cell-mediated destruction of hair follicles, leading to distinct patches of nonscarring alopecia on any body part. The pathogenesis of AA is complex, involving genetic, epigenetic, immunologic, gut and skin microbiome, allergy, and oxidative stress factors [18,19]. The most commonly affected areas are the scalp and beard, the latter constituting approximately 28% of cases [20]. Onset typically occurs between 30 and 40 years old, with a recurrence rate reported between 15 and 25.5%. Alopecia areata of the beard (AAB) may be associated with various conditions such as diabetes mellitus, vitiligo, hypothyroidism, and vitamin D deficiency [20,21,22]. 

Considering the aforementioned information, the objective of this review is to perform an in-depth analysis of the relevant specialty literature concerning the common skin affections observed in APECED. This includes the signature cutaneous manifestation, chronic mucocutaneous candidiasis, alopecia areata, and vitiligo while also offering a concise overview of their clinical presentations.

## 2. Materials and Methods

This paper is a narrative review of specialty literature published in English from inception to October 2023. We included a total of 14 original human studies that were clinically significant. These studies were identified using several combinations of specific keywords, including: “chronic mucocutaneous candidiasis”, “APECED APS-1”, “Vitiligo”, and “Alopecia” in the PubMed research database. We specifically searched for studies that described chronic mucocutaneous candidiasis reported in patients with APECED, alopecia, and vitiligo reported in individuals with APECED. 

For the “chronic mucocutaneous candidiasis & APECED” and “chronic mucocutaneous candidiasis & APS-1” combinations, we identified a total of 88 and 31 results, respectively. 

In the combinations of “Alopecia & APECED” we found a total of 22 results, while in the combinations of “Alopecia & APS-1”, we found 2 results. 

For the “Vitiligo & APECED” and “Vitiligo & APS-1” combinations, a total of 35 and 3 results, respectively were detected. 

We identified a total of 181 publications, which were then subjected to manual searching to align with the goals of our study. We selected studies that focused on populations comprising varying numbers of individuals diagnosed with APECED, with sample sizes ranging from 7 to 158 patients. Reviews, meta-analyses, or case reports were excluded from this paper.

## 3. Results

### 3.1. Cutaneous Manifestations in APECED

#### 3.1.1. Chronic Mucocutaneous Candidiasis 

Chronic mucocutaneous candidiasis (CMC) is a condition characterized by recurrent or persistent symptomatic infections of the mucocutaneous tissues, typically caused by *Candida species*, *Candida albicans* being the particularly predominant pathogen [23,24]. The diagnosis of CMC is primarily clinical, often accompanied by the isolation of *Candida* spp. from the affected body sites. Opportunistic mucosal infections, deep organ infections, or systemic infections tend to occur in immunocompromised patients and usually arise from Candida colonization in the digestive tract [23,24]. It presents in various forms, from mild oral symptoms such as ulceration and soreness to more severe forms affecting the entire oral cavity, making it difficult to consume certain foods. Additionally, CMC can affect the nails and, to a lesser extent, the skin [25]. In most APS I patients, chronic mucocutaneous candidiasis is a common early manifestation that appears before the associated endocrine features [26]. This condition is characterized by recurrent or persistent fungal infections affecting mucous membranes, skin, and nails. Oral candidiasis is most prevalent during the first two years of life and often takes a chronic course [27]. Cutaneous candidal infections can lead to conditions such as intertrigo, angular cheilitis, scalp infections, and diaper or perianal candidiasis, while nail fungal infections often result in nail discoloration, thickening, and paronychia [28,29].

This pathology represents the, signature’ manifestation of APS-1, hence its typically high occurrence rate in these patients. This data is supported by multiple studies conducted on different populations. Results have shown that chronic mucocutaneous candidiasis was exhibited in APS-1 patients from Finnish, Irish, Saudi Arabian, Italian, North American, Croatian, and Norwegian populations, with a prevalence between 51.3% and 100% [13,30,31,32,33,34,35,36,37,38,39,40,41,42]. A retrospective study conducted by Ahonen et al. between 1910–1988 on 68 Finnish patients previously diagnosed with APECED, showed that 100% had a type of CMC [30]. Also, another Finnish population showed a 60% prevalence of CMC among individuals diagnosed with APS-1, in a cross-sectional study by Perheentupa in 2006 [33]. In Ireland, a cross-sectional study of 18 APS-1 patients conducted by Collins et al. revealed 100% of patients experiencing CMC, 72% of those presenting candidal onychomycosis or paronychia. In contrast, a study conducted by Domingues et al. showed a prevalence of 80% [34,35]. A retrospective survey of siblings from consanguineous families in Saudi Arabia found that 14 out of the 20 siblings with APS-1 exhbited CMC [37]. Additionally, an observational study conducted by Proust-Lemoine et al. on a French population of 19 individuals with APS-1 showed CMC occurred in 17 individuals [36]. A prospective study conducted by Ferre et al. on a North American population of 35 APS-1 patients showed that the prevalence of CMC increased with age, ranging from 51% around 1 year to 86% at around 65 years and above [13]. Moreover, an increased occurrence with age was also shown in a prospective study on 158 Italian patients conducted by Garelli, with 51.3% at onset and 74.7% at the end of the follow-up [41]. Finally, a recent observational study conducted on 7 Croatian patients with APS-1 by Skrabic et al., showed a prevalence of 57.1% of CMC [42]. In contrast, a survey conducted by Zlotogora et al. in 1992 on a Jewish Iranian population of 23 patients with APS-1 identified a significantly lower prevalence of CMC (17%) [31]. 

Chronic mucocutaneous candidiasis may also occur as a first sign in individuals with APECED. In Italian populations, studies conducted by Meloni et al. and Betterle et al. concluded that this pathology presented as a first sign in 18 out of 21 patients and 93% of cases, respectively [32,38]. It is crucial to closely monitor individuals, especially children, presenting with isolated CMC, using immunological, biochemical, and clinical assessments to detect potential endocrine glandular affections [25].

As per the female/male ratio, a prospective study on a Norwegian population of 52 patients from 34 families with APS-1, showed that from a total of 40 out of 52 (77%) diagnosed with CMC, 15 were females, and 25 were males [39].

In almost all of these studied populations, chronic mucocutaneous candidiasis tended to appear early, with the mean age ranging from 4.4 years in the American population to 9.1 years in the Italian population [13,41].

According to Ferre et al. and Ahonen et al., the most frequent site affected by CMC was the oral cavity, with a prevalence of 100% in both studied populations, followed by vulvovaginal candidiasis (52.4% of the female individuals), esophageal candidiasis (51.4%), ungual candidiasis (34.3% to 71%) and, finally, dermal candidiasis (9% to 17.1%) [13,30]. Results have been presented in Table 1.

#### 3.1.2. Alopecia and Vitiligo in APECED

Alopecia areata (AA) stands as an autoimmune disorder characterized by nonscarring hair loss. This condition hinges on autoimmunity, a complex immunologic response pattern wherein the immune system erroneously targets “normal” tissue [43,44].

Within this framework, AA takes on the form of an immune-mediated disorder, and it is most frequently observed in individuals with APECED. AA is fundamentally hypothesized to be an organ-specific autoimmune ailment where T cells play a central role in attacking hair follicles [45,46].

Though most AA cases manifest sporadically, a growing body of evidence suggests that it represents a multifaceted, multi-genetic trait marked by inherited predisposition. The manifestation of AA in APS-1 patients remains relatively understudied, and it’s important to note that, as per Hedstrand et al., the most prevalent form within APS-1 patients is alopecia totalis or universalis [47].

Further reinforcing the significance of understanding this condition’s genetic and autoimmune dimensions, Tazi-Ahnini et al. reveal that while the general population has a lifetime risk of approximately 1.4%, in the context of APECED, this risk of developing AA is increased to over 30% [48]. 

The studies that we reviewed in this paper (Table 2) showed that AA was not present in the majority of the individuals, with a prevalence ranging from 13% in an Iranian Jewish population [31] to 45% in a Saudi Arabian population [37]. In a Finnish population, Ahonen et al. concluded that the prevalence of AA was 29%. Betterle et al. identified an AA prevalence of 37% in an Italian population, and Collins et al. showed a prevalence of 33% in an Irish population [30,32,35].

In the North and South American population, Ferre et al. described an increasing prevalence of AA with age, ranging from 0% in individuals being 1 year of age to 17% in patients 60+ years old, with mean age 8 years old [13].

The female/male ratio was addressed in the prospective study conducted by Bruserud et al. on the Norwegian population, with the prevalence of AA being 31% (16 patietns/25), out of which 5 were females and 11 were males [39]. 

Additionally, in the Italian population investigated by Meloni et al., AA occurred in three males, and its manifestation varied from alopecia areata to alopecia totalis; in the French population studied by Proust-Lemoine et al., AA was identified in 10 out of 19 individuals, 2 presenting alopecia universalis [36,38].

Vitiligo, a prevalent depigmenting skin disorder affecting approximately 0.5–2% of the global population, is often associated with polyendocrinopathies [49,50,51]. While the exact cause of vitiligo remains elusive, there is compelling indirect evidence linking anti-melanocyte antibodies to the development of this condition in both animal and human cases [52,53,54]. This common depigmenting disorder is characterized by the distinctive loss of melanocytes, resulting in the appearance of chalky-white macules that are typically non-scaly in nature [49,55].

In recent years, significant strides have been made in our comprehension of the pathogenesis of vitiligo, leading to its precise classification as an autoimmune disease [49,56].

The prevalence of vitiligo among individuals with APS-1, presented in the studies we reviewed in this paper, ranged between 11.1% in the Irish population and 37.1% in the North and South American population [13,35]. Meloni et al. identified 2 cases of vitiligo in the female population, with manifestations varying from spots to extensive depigmentation of skin and hair, in the Sardinian population, while Proust-Lemoine et al. identified 4 out of 19 cases of vitiligo, one female presenting universal vitiligo [36,38]. According to Ferre et al. and Garelli et al., vitiligo cases tended to be higher with age, ranging from 0% in individuals of 1 year of age to 37% in individuals 60+ years old in the North and South American population and from 2% at onset to 17% at the end of the follow-up in the Italian population [13,41]. In addition, in the Norwegian population, Bruserud et al. showed that the vitiligo prevalence was 15% (8 out of 52 patients), affecting both genders equally (4 females and 4 males) [39].

#### 3.1.3. Other Cutaneous Findings in APECED

Over the past few decades, more than 30 distinct autoimmune manifestations have been documented, with specific disease components exhibiting variable representation in different patient cohorts. Notably, more than 25 of these manifestations affect non-endocrine tissues [57,58,59]. 

As shown in Table 3, the North and South American population studied by Ferre et al. identified an urticarial rash in 78.6% of the individuals with APS-1, being the most prevalent initial manifestation among all patients, in conjunction with chronic mucocutaneous candidiasis (CMC), with a mean debut age 1.6 years. These urticarial eruptions typically appeared as either flat or, more commonly, as a raised erythematous rash that extended over a wide area, manifesting within a range of 3 to 36 months. In 90.9% of the cases, the rash was non-pruritic, and in 72.2% of the cases it was non-blanching. It often occurred on the torso at first, then continued to spread to the extremities and the face but sparing soles and palms. It cleared up after an average of 146.8 days, but it recurred in 72.7% of cases, presenting the same features. The diagnosis of urticarial rash was suggested by the histopathological examination which showed mild/moderate perivascular inflammation in both deep and superficial dermis, patchy lymphoid and myeloid infiltrates including among others T lymphocytes, eosinophils, neutrophils with associated karyorrhexis and plasma cells. Moreover, vacuolar changes at the dermal-epidermal junction and lobular-pattern panniculitis were observed [13]. Additionally, Perheentupa et al. identified that in the Finnish population, 13 patients out of 91 had an urticarial rash. A skin biopsy was conducted in four cases, revealing that two exhibited lymphoplasmacytic vasculitis [33].

### 3.2. Endocrine Manifestations in APECED 

#### 3.2.1. Chronic Hypoparathyroidism and Addison’s Disease

Patients diagnosed with autoimmune polyendocrinopathy-candidiasis-ectodermal dystrophy (APECED) often experience the classic triad of hypoparathyroidism, chronic mucocutaneous candidiasis, and Addison’s disease, with the presence of at least two of these components being diagnostic. Typically, one of the first disease components to appear is hypoparathyroidism, which usually manifests in childhood. However, the clinical presentation of APECED is quite heterogeneous, making it challenging to diagnose. As a result, APECED is typically not diagnosed until after the first decade of life [60,61]. The clinical studies examined in this paper revealed a prevalence of chronic hypoparathyroidism (CH) exceeding 50%, ranging from 73% in the Norwegian population to as high as 96% in the Iranian Jewish population [31,39]. Additionally, in the Norwegian population, CH was the initial manifestation in 32% of the cases [39]. In contrast, Perheentupa et al. identified a prevalence of only 32% in the Finnish population, and Garelli et al. showed a prevalence of 34.8% of CH at the beginning of the study, but it increased to 86.1% at the end of the follow-up [33,41]. Furthermore, Skabic et al. identified a CH prevalence of 42.9% in the Croatian population [42]. Meloni et al. identified a CH prevalence of 77% in Sardinian individuals, females representing 92.3% of the cases and males 62.5% with a *p*-value of 0.043. Moreover, the debut of this affection was earlier in females, the age range being 3–9 years, in contrast to the male age range which was 4.5–20 years, with a *p*-value of 0.015 [38]. Moreover, Bruserud et al. identified a female/male ratio of 18/20 in the Norwegian population and a median age at onset of 9 years [39]. According to Ferre et al., the prevalence of chronic hypoparathyroidism (CH) demonstrated a tendency to increase with age, varying from 6% at around 1-year-old to 91% in individuals aged 60 years and older [13].

Primary adrenal insufficiency (PAI) is a rare condition, with a current prevalence in Western societies ranging from about 100 to 140 cases per million [62]. Notably, this reported prevalence has shown a significant increase over time, as it was in the range of 40 to 70 cases per million in Europe during the 1960s [63]. Intriguingly, recent data indicate a continuation of this upward trend, with a further rise in the prevalence of PAI, particularly among women [64]. AD is characterized by a relatively low incidence, with autoimmune causes being the predominant factor, especially in developed countries. It can manifest as an isolated condition, but over 50% of cases are associated with other autoimmune disorders, including APS-1. The dynamic nature of these disorders emphasizes the significance of continuous research and clinical vigilance in comprehending and effectively managing these intricate autoimmune endocrine conditions [65,66]. AD is part of the classic triad of APS-1, thus most of the studies we reviewed in this paper, identified AD with prevalence of over 50% ranging from 63% in the Norwegian population studied by Bruserud et al. to 83% in the North and South American population studied by Ferre et al. [13,39]. Conversely, Zlotogora et al. identified an AD prevalence of 21.7% in the Iranian Jewish population, and Perheentupa et al. identified a prevalence of 5% in the Finnish population. In contrast, in the Saudi Arabian population studied by Bin-Abbas, only 8 out of 20 individuals had AD [31,33,37]. In the Sardinian population, Meloni et al. detected an AD prevalence of 68%, this pathology presenting as the first endocrinopathy in 5 individuals out of the 22 studied [38]; moreover, according to Bruseurd et al. AD was detected in 33 out of 52 patients (63%), 11 being females and 22 males, with a median age of onset of 13 years [39]. Furthermore, it appeared that Addison’s disease (AD) exhibited an increasing prevalence with advancing age. According to Ferre et al., the prevalence of AD ranged from 0% in individuals at the age of 1 to 83% in patients aged 60 and above [13]. Also, Garelli et al. determined that the initial prevalence of Addison’s disease (AD) was 17%, and it increased to 77.2% at the end of the follow-up period. In 6.3% of cases, AD occurred as a standalone condition, whereas in 10.7% of cases, AD was found in conjunction with other pathologies [41]. An observational study conducted by Skrabic et al. on 7 Croatian patients found that 100% of them had either CH or AD, with 14.2% of patients having five endocrinopathies, another 14.2% having four endocrinopathies, and 28.7% having three endocrinopathies. Furthermore, 14.2% had two endocrinopathies, while an additional 28.7% had just one endocrinopathy [42]. Results have been summarized in Table 4.

#### 3.2.2. Classic Triad in APECED

Autoimmune polyglandular syndrome type 1 is characterized by a classic triad of hypoparathyroidism, adrenal insufficiency, and candidiasis as a result of homozygous loss-of-function mutations in the AIRE gene [60,67]. In some of the studied populations we reviewed in this article, most individuals presented the classic triad of APS-1, ranging from 51% in the Finnish population to 67% in the Norwegian population [30,39]. Additionally, in the North and South American population, 20% presented only two of the components, CMC and CH, respectively, while in the Norwegian population, 9 out of 19 had the classic triad, 7 out of 19 presented with a diagnostic dyad, and 3 out of 19 had only one component of the three key manifestations [13,36]. Furthermore, individuals with APECED also experience a range of non-endocrine autoimmune symptoms with varying frequencies. Identifying these manifestations by healthcare providers can expedite the diagnostic process and enable the timely initiation of specific screening, preventative, and treatment measures [3,60]. All of the results are summarized in Table 5.

## 4. Discussion

The skin often becomes a valuable tool for clinicians, offering insights into understanding, diagnosing, and monitoring endocrine diseases. Dermatologic signs of endocrine disorders play a crucial role in an individual’s overall health and quality of life [27,68]. The wide-ranging clinical presentation and course of APECED is a significant observation. Recognizing the diversity in early clinical manifestations is vital for early diagnosis. While the classic diagnostic dyad is definitive when present, its absence lacks diagnostic value [33,69,70]. Such pathologies as chronic oral candidiasis, a well-known APECED indicator, should raise suspicion of the condition if it appears after the newborn period. However, in the Finnish population studied by Perheentupa et al. it was not present in over two-thirds of Finnish patients [33]. Conversely, among 24 Iranian Jewish patients with their unique mutation, only four had candidiasis [31]. Moreover, various early signs, such as periodic rash with fever, alopecia, and vitiligo, should be recognized as potential indicators of APECED. 

Most studies examined in this paper have consistently demonstrated the early onset of chronic mucocutaneous candidiasis (CMC), alopecia, and vitiligo within the populations under analysis. This underscores the critical importance of conducting meticulous and thorough monitoring when confronted with these pathologies. Notably, in the Saudi Arabian cohort, CMC manifested during the neonatal period, while in the Sardinian population, it emerged as the primary manifestation of APECED [37,38]. Furthermore, the investigation by Ahonen et al. on the Finnish population revealed instances where CMC served as the exclusive presenting sign [30]. Additionally, the Croatian population exhibited onychodystrophy as the initial manifestation in a substantial number of individuals. Thus, the identification of onychodystrophy as a potential new warning sign for APECED syndrome adds a layer of complexity to its diagnostic landscape, further highlighting the need for a comprehensive understanding of its diverse manifestations [42]. In the Irish population, the diagnosis was established upon the identification of dermatological manifestations and subsequently confirmed through mutational analysis in two of the included patients. However, alopecia areata and vitiligo manifested at later stages, possibly indicating a more pronounced disease severity in these instances [35]. In contrast, the Norwegian population experienced delays in the diagnostic process due to atypical late presentations and protracted intervals between the initiation of various clinical components [39].

Nonetheless, the studies included in this review exhibited noteworthy limitations, such as modest sample sizes, thereby instigating apprehensions regarding the extrapolation of findings to broader demographic cohorts. Additionally, an incomplete and non-systematic examination of various symptoms was observed, introducing constraints on the study’s capacity to encompass the entire spectrum of the disease. The underestimation of true incidence, inadequate mortality data, susceptibility to biases stemming from temporal alterations in population dynamics, survival and detection methodologies, alongside potential biases in accentuating early component manifestations, further underscored the limitations. Consequently, future research endeavors should systematically address these constraints to fortify the robustness and generalizability of outcomes.

Several cases have been reported in literature that support the importance of recognizing potential signs of APS-1 to insure prompt diagnosis. Carboni et al. reported a case of a 37-year-old diagnosed with nail candidiasis of both fingernails and toenails since the age of 8. After five years, AD and CH occurred, thus confirming the diagnosis of APS-1. This patient later underwent replacement therapies to address endocrinological deficiencies and received systemic itraconazole for a two-month period of time. This intervention led to a significant amelioration of Candida infections [71]. Furthermore, Minka et al. reported two cases: one of a four-year-old boy who was presenting chronic cutaneous candidiasis since five months of age and was later diagnosed with APS-1 and one of a thirteen-year-old girl who was referred by a dermatologist for endocrinology review for diffuse and repeated cutaneous candidiasis since five months of age. The diagnosis of APS-1 was also set in this patient [72]. As also indicated by several aforementioned authors, mucocutaneous candidiasis served as the inaugural manifestation in these patients.

In contrast, Bhansali et al. reported the case of a 16-year-old boy who presented with an adrenal crisis. Hashimoto’s thyroiditis, alopecia, and subnormal T-cell function were identified during the evaluation. Subsequently, a diagnosis of APECED was made, even though chronic mucocutaneous candidiasis was not observed [73]. Additionally, Improda et al. described the case of a seven-month-old female who presented with a vasculitic rash that consisted of multiple purple-colored plaques with irregular margins localized on the trunk and limbs. By the age of five, she was presenting recurrent cutaneous candidiasis, alopecia, autoimmune thyroiditis, and various digestive symptoms. Eventually, the diagnosis of APECED was confirmed, although delayed, through genetic testing [74]. This case underscores the imperative for vigilant monitoring of children displaying unexplained vasculitic skin rash, facilitating the timely recognition of (APS-1).

In individuals afflicted with APECED, the etiology of extensive autoimmunity is predicated upon the thymic escape of autoreactive T cells, culminating in the manifestation of diverse endocrinopathies and the development of autoantibodies targeting interleukin-17 (IL-17) and type I interferons (IFNs). The management of this syndrome poses considerable challenges, characterized by the absence of a definitive cure and the deficiency of a substantial preventive or mitigating strategy for autoimmune manifestations. Poorly treated or underdiagnosed endocrinologic pathologies can be fatal [39]. Existing therapeutic approaches predominantly hinge upon hormonal substitution regimens aimed at ameliorating the consequences of endocrinopathies [12,75,76,77].

Additionally, recently documented in the literature is the noteworthy and clinically substantial influence of the Janus kinase (JAK) inhibitor, ruxolitinib, on a myriad of manifestations associated with APS-1. These included AA, CMC, nail dystrophy, keratitis, autoimmune hepatitis, hypoparathyroidism, diabetes insipidus, renal potassium loss, and exocrine pancreatic insufficiency. The treatment demonstrated a positive impact on the patients’ quality of life, with sustained effectiveness on manifestations observed even after a 30-month follow-up period, and no biological or infectious adverse effects were observed [78].

The intricate interplay of immunological dysregulation in APS-1 necessitates comprehensive investigations for the elucidation of underlying pathogenic mechanisms, prompting a critical imperative for the development of targeted interventions to modulate the autoimmune cascade in affected individuals.

The complex interaction between the endocrine and integumentary systems often leads to dermatologic pathologies when endocrine hormones become dysregulated in various endocrinopathies. Recognizing these skin manifestations is vital for clinicians since they can serve as valuable indicators of underlying endocrine problems and significantly impact a patient’s health [27]. Further research is necessary to fully elucidate the genotype-phenotype relationship in more extensive international populations accompanied by extended follow-up periods for APECED patients. In addition, more investigations should specifically focus on identifying potential yet undetected initial disease manifestations.

## 5. Conclusions

In conclusion, it is noteworthy that APECED poses a diagnostic challenge and remains a subject of ongoing discussion due to its rare occurrence and the intricate array of clinical presentations.

The genetic and autoimmune dimensions of these cutaneous manifestations underscore the complexity of APECED. Understanding these skin affections has improved over the years, with research shedding light on the role of IL-17-mediated immunity in mucocutaneous defense against *Candida* spp. Furthermore, the prevalence of vitiligo and AA in APECED individuals is influenced by a combination of genetic predisposition and autoimmune mechanisms.

Based on a thorough analysis of specialty literature, this review highlights the clinical presentations and prevalence of cutaneous manifestations in APECED, emphasizing the importance of early detection and comprehensive monitoring. The classic triad remains a crucial diagnostic feature, but the heterogeneity of clinical presentations, including cutaneous manifestations, necessitates a multidimensional approach to identify and manage APECED accurately. Ongoing research and clinical vigilance are essential further to unravel the intricacies of this rare autoimmune syndrome and enhance patient care.

## Figures and Tables

**Table 1:** Chronic mucocutaneous candidiasis in APECED.

First Author/Year	Type of Study	Studied Population	Time Frame	Chronic Mucocutaneous Candidiasis
Ahonen /1990 [30]	Retrospective	N = 68 Finnish patients with APS-1	1910–1988	oral candidiasis: N = 68 (100%) ungual candidiasis: N = 48 (71%) dermal candidiasis: N = 6 (9%) 1month-21 years
Zlotogora /1992 [31]	Observational	N = 23 patients N1 = 2 females N2 = 11 males from 19 families of Iranian Jewish community APS-1	N/A	N = 4 (17%)
Betterle /1998 [32]	Prospective	N = 41 patients with APS-1 (15 from Veneto, 9 from the area of Vicenza)	1967–1996	N = 1
Perheentupa/2006 [33]	Cross-sectional	N = 91 Finnish patients wth APS-1	N/A	60% esophageal canddiasis: N = 14
Dominguez/2006 [34]	Observational	N = 31 Irish patients with APS-1 N1 = 18 females N2 = 13 males ages 2–56 years from 19 families giving an Irish prevalence of 1:130,000	N/A	N = 25 (80%)
Collins /2006 [35]	Cross-sectional	N = 18 Irish patients with APS-1 N1 = 11 females, M2 = 7 males	N/A	100% candidal onychomycosis or paronychia: N = 13 (72%)
Proust-Lemoine /2010 [36]	Observational	N = 19 French patients with APS-1	2007–2008	N = 17
Bin-Abbas /2010 [37]	Retrospective	N = 20 siblings with APS-1 from 7 consanguineous families in Saudi Arabia	6 years	N = 14
Meloni /2012 [38]	Prospective	N = 22 Sardinian pediatric patients with APS-1 from 17 families N1 = 13 females N2 = 9 males median age, 30.7 years; (range, 1.8–46 years)	1984–2011	95% first sign: N = 18 (86%) median age 3.0 years, (range, 3 months to 10 years) varied from recurrent oral/ungual to lifelong oral candidiasis Candida esophagus: N = 2 esophageal stricture: N = 1
Ferre/2016 [13]	Prospective	N = 35 consecutive patients from 32 nonconsanguineous families with clinical and/or genetic diagnosis of APS-1 from North and South America N1 = 1 female N2 = 14 males mean age 20 years (range, 7–64 years); N3 = 16 children 16 (45.7%) children, with a mean age of 11.6 years	2013–2015	86% oral thrush 100% vulvovaginal candidiasis 52.4% of females esophageal candidiasis 51.4%. nail candidiasis. 34.3%. cutaneous candidiasis 17.1% 1 year 51% 2 years 57% 5 years 63% 10 years 74% 15 years 80% 20 years 80% 30 years 86% 40 years 86% 50 years 86% 60+ years 86% Age range (median, mean) 0.5–26 (1, −4, 4)
Bruserud/2016 [39]	Prospective	N = 52 patients from 34 Norwegian families with APS-1	1996–2016	N = 40 (77%) Females/Males 15/25 Median Age at Onset, years 7, 5 (0–64). 25% initial manifestation 15% Candida esophagitis. 1 patient stenosis of the esophagus 1 patient severe Candida otitis
Saari/2021 [40]	Cross-sectional cohort study combined with longitudinal follow-up data	N = 19 Finnish patients (all females) with APS-1 median age of 42.6 years (range, 16.7–65.5)	N/A	21% (genital infection)
S Garelli/2021 [41]	Prospective	N = 158 Italian patients with APS-1 N1 = 103 females N2 = 55 males	at the onset and during a follow-up of 23.7 ± 15.1 years	Onset 51.3% mean age of 4.4 ± 6.4 years End of follow-up 74.7% mean age of 9.1 ± 13.6 years
Skrabic/2022 [42]	Observational	N = 7 Croatian patients with APS-1 N1 = 3 females N2 = 4 males (mean current age of 25.3 years (age range from 5.4 to 40.2 years))	37 years	57.1%

N/A = Not Applicable.

**Table 2:** Alopecia and Vitiligo in APECED.

First Author/Year	Type of Study	Studied Population	Time Frame	Cutaneous Manifestations	
				Alopecia Areata	Vitiligo
Ahonen/1990 [30]	Retrospective	N = 68 Finnish patients with APS-1	1910–1988	N = 20 (29%) 4 months–21 years	N = 9 (13%) 1 month–15 years
Zlotogora/1992 [31]	Observational	N = 23 patients (11 males, 12 females) from 19 families of Iranian Jewish community APS-1	N/A	N = 3 (13%)	N/A
Betterle/1998 [32]	Prospective	N = 41 patients with APS-1 (15 from Veneto, 9 from the area of Vicenza)	1967–1996	N = 15 (37%)	N = 6 (15%) complement-fixing melanocyte autoantibodies: N = 5
Perheentupa/2006 [33]	Cross-sectional	N = 91 Finnish patients wth APS-1	N/A	N = 11	N/A
Collins/2006 [35]	Cross-sectional	N = 18 Irish patients (11 females, 7 males) with APS-1	N/A	N = 6 (33%)	N = 2 (11.1%)
Proust-Lemoine/2010 [36]	Observational	N = 19 French patients with APS-1	2007–2008	N = 10 alopecia universalis: N = 2	N = 4 universal vitiligo: N = 1 (female)
Bin-Abbas/2010 [37]	Retrospective	N = 20 siblings with APS-1 from 7 consanguineous families in Saudi Arabia	6 years	N = 9 (45%)	N/A
Meloni/2012 [38]	Prospective	N = 22 Sardinian pediatric patients with APS-1 from 17 families N1 = 13 females N2 = 9 males (median age, 30.7 years; range, 1.8–46 years)	1984–2011	Alopecia: N2 = 3 males (varied from areata to totalis) Vitiligo: N1 = 2 females (varied from spots to extensive depigmentation of skin and hair) more common after age 15 years (range, 8–25 years)	
Ferre/2016 [13]	Prospective	N = 35 consecutive patients from 32 nonconsanguineous families with clinical and/or genetic diagnosis of APS-1 from North and South America N1 = 14 males N2 = 1 female mean age 20 years (range, 7–64 years); N3 = 16 (45.7%) children, with a mean age of 11.6 years	2013–2015	17.1% 1 year 0% 2 years 3% 5 years 6% 10 years 11% 15 years 17% 20 years 17% 30 years 17% 40 years 17% 50 years 17% 60+ years 17% Age range (median, mean) 2–15 (8–8)	37.1%. 1 year 0% 2 years 3% 5 years 9% 10 years 17% 15 years 23% 20 years 31% 30 years 31% 40 years 34% 50 years 34% 60+ years 37% Age range (median, mean). 2–63 (11–15, 5)
Bruserud/2016 [39]	Prospective	N = 52 patients from 34 Norwegian families with APS-1	1996-2016	N = 16 (31%) Females/Males 5/11 Median Age at Onset 19 (4–41)	N = 8 (15%) Females/Males 4/4 Median Age at Onset 20 (15–51)
S Garelli/2021 [41]	Prospective	N = 158 Italian patients with APS-1 N1 = 103 females N2 = 55 males	at the onset and during a follow-up of 23.7 ± 15.1 years	Onset 2% 3 years of age End of follow-up 24% mean age of 13 ± 10 years	Onset 2% mean age of 4 ± 1.7 years End of follow-up 17% mean age of 17 ± 15 years

N/A = Not Applicable.

**Table 3:** Other cutaneous findings in APECED.

First Author/Year	Type of Study	Studied Population	Time Frame	Urticarial Rash
Ferre/2016 [13]	Prospective	N = 35 patients from North and South America	2013–2015	78.6%
Perheentupa/2006 [33]	Cross-sectional	N = 91 Finnish patients wth APS-1	N/A	N = 13 lymphoplasmacytic vasculitis: N = 2

N/A = Not Applicable.

**Table 4:** Main endocrine manifestations in APECED.

First Author/Year	Type of Study	Studied Population	Time Frame	Endocrine Manifestations	
				Chronic Hypoparathyroidism (CH)	Addison’s Disease (AD)
Ahonen/1990 [30]	Retrospective	N = 68 Finnish patients with APS-1	1910–1988	79% (19 months–44 years old)	72% (4.2 to 41 years)
Zlotogora/1992 [31]	Observational	N = 23 patients N1 = 12 females N2 = 11 males from 19 families of Iranian Jewish community APS-1	N/A	N = 22 (96%)	N = 5 (21.7%)
Betterle/1998 [32]	Prospective	N = 41 patients with APS-1 (15 from Veneto, 9 from the area of Vicenza)	1967–1996	N = 38 (93%) mean age of onset was 9.2 years (range, 2–36 years)	N = 30 (73%) mean age of 13.6 years (range, 2–37 years)
Perheentupa/2006 [33]	Cross-sectional	N = 91 Finnish patients with APS-1	N/A	32%	5%
Dominguez/2006 [34]	Observational	N = 31 Irish patients with APS-1 N1 = 18 females N2 = 13 males ages 2–56 years from 19 families giving an Irish prevalence of 1:130,000	N/A	N/A	N = 21 (67%)
Proust-Lemoine/2010 [36]	Observational	N = 19 French patients with APS-1	2007–2008	N = 13	N = 15
Bin-Abbas/2010 [37]	Retrospective	N = 20 siblings with APS-1 from 7 consanguineous families in Saudi Arabia	6 years	N/A	N = 8
Meloni/2012 [38]	Prospective	N = 22 Sardinian pediatric patients with APS-1 from 17 families N1 = 13 females N2 = 9 males, median age, 30.7 years; (range, 1.8–46 years)	1984–2011	77% females 92.3% vs. males 62.5%; *p* = 0.043 age range: females 3–9 years vs. males 4.5–20 years; *p* = 0.015	68% First endocrinopathy: N = 5 median 9 years
Ferre/2016 [13]	Prospective	N = 35 consecutive patients from 32 nonconsanguineous families with clinical and/or genetic diagnosis of APS-1 from North and South America N1 = 1 females N2 = 14 males mean age 20 years (range, 7–64 years); N3 = 16 (45.7%) children, with a mean age of 11.6 years	2013–2015	85–90% 1 year 6% 2 years 29% 5 years 57% 10 years 83% 15 years 86% 20 years 89% 30 years 91% 40 years 91% 50 years 91% 60+ years 91% Age range (median, mean) 0, 5–29 (5–5, 9)	85–90% 1 year 0% 2 years 6% 5 years 23% 10 years 54% 15 years 71% 20 years 77% 30 years 80% 40 years 83% 50 years 83% 60+ years 83% Age range (median, mean) 2–34 (8–9, 9)
Bruserud/2016 [39]	Prospective	N = 52 patients from 34 Norwegian families with APS-1	1996–2016	N = 38 (73%) Females/Males 18/20 Median Age at Onset, years 9 (1–60) 32% initial manifestation	N = 33 (63%) Females/Males 11/22 Median Age at Onset 13 (4–55)
Saari/2021 [40]	Cross-sectional cohort study combined with longitudinal follow-up data	N = 19 Finnish patients (all females) with APS-1 median age of 42.6 years (range, 16.7–65.5)	N/A	N/A	79%
S Garelli/2021 [41]	Prospective	N = 158 Italian patients with APS-1 N1 = 103 females N2 = 55 males	at the onset and during a follow-up of 23.7 ± 15.1 years	Onset 34.8% mean age of 8.4 ± 10.2 years isolated CH 21.5% CH associated with other diseases 13.3% End of follow-up 86.1% mean age of 11.1 ± 12.1 years	Onset 17% mean age of 18.8 ± 18.6 years isolated AD 6.3% AD associated with other diseases 10.7% End of follow-up 77.2% mean age of 16.3 ± 14.1 years
Skrabic/2022 [42]	Observational	N = 7 Croatian patients with APS-1 N1 = 3 females N2 = 4 males mean current age of 25.3 years (age range from 5.4 to 40.2 years)	37 years	100% had CH or AD 42.9% CH 14.2% five endocrinopathies 14.2% four endocrinopathies 28.7% three endocrinopathies 14.2% two endocrinopathies 28.7% one endocrinopathy	

N/A = Not Applicable.

**Table 5:** Classic triad findings.

First Author/Year	Type of Study	Studied Population	Time Frame	Results
Ahonen/1990 [30]	Retrospective	N = 68 Finnish patients with APS-1	1910–1988	classic triad: N = 35 Age range 4.5 to 44 years Mean age 14
Meloni/2012 [38]	Prospective	N = 22 Sardinian pediatric patients with APS-1	1984–2011	classic triad: 58%
Ferre/2016 [13]	Prospective	N = 35 patients from North and South America	2013–2015	CMC and CH: N = 7 (20%) classic triad: N = 22 (62.9%)
Bruserud/2016 [39]	Prospective	N = 52 patients from 34 Norwegian families with APS-1	1996–2016	classic triad: N = 4 (67%) developed by 25 years of age
Proust-Lemoine/2010 [36]	Observational	N = 19 French patients with APS-1	2006–2008	classical triad: N = 9 dyad: N = 7 one component of the triad: N = 3
